# Typhoid fever: issues in laboratory detection, treatment options & concerns in management in developing countries

**DOI:** 10.4155/fsoa-2018-0003

**Published:** 2018-06-26

**Authors:** Balaji Veeraraghavan, Agila K Pragasam, Yamuna D Bakthavatchalam, Ravikar Ralph

**Affiliations:** 1 Department of Clinical Microbiology, Christian Medical College, Vellore 632004, Tamil Nadu, India; 2 Department of Medicine, Christian Medical College, Vellore 632004, Tamil Nadu, India

**Keywords:** azithromycin, cefixime, ceftriaxone, *Salmonella* Typhi

## Abstract

Multidrug-resistant *Salmonella enterica* subsp. *enterica* serovar Typhi (resistant to ampicillin, chloramphenicol and cotrimoxazole), was significantly reduced with the increased usage of fluoroquinolones and azithromycin. This has led to declining multidrug resistance rates in India with increasing ciprofloxacin nonsusceptibility rates and clinical failures due to azithromycin. However, for the available agents such as ceftriaxone, azithromycin and fluoroquinolones, the dose and duration for treatment is undefined. The ongoing clinical trials for typhoid management are expected to recommend the defined dose and duration for better clinical outcome. We made an attempt to summarize the issues in laboratory detection, treatment options and responses, and the concerns in clinical practice seen in the developing countries.

## Lay abstract

Typhoid fever is an important cause of mortality in developing countries and is a major public health concern. Cephalosporins or azithromycin are the drugs of choice for treating infection caused by the reduced fluoroquinolone susceptibility of *S.* Typhi. Emergence of cephalosporin resistance in *S.* Typhi and azithromycin-associated clinical and microbiological failure is of significant concern in developing countries. An approach of cephalosporin–azithromycin combination therapy has been suggested, which could be a potential alternative in treating uncomplicated *S.* Typhi infection in endemic areas. This review summarizes the field so far.

Typhoid fever is caused by the bacterium *Salmonella enterica* subsp. *enterica* serovar Typhi. It is mainly due to the inadequate access to safe water and sanitation, which is a major problem in developing countries. The global burden of typhoid fever was estimated to be 12 million cases and 130,000 deaths in the year 2010. It exceeded 100 cases per 100,000 people/year in South East Asian countries, and has especially high burden rates in India. A recent systematic review and meta-analysis estimated the prevalence of laboratory confirmed typhoid and paratyphoid cases in India to be 9.7 and 0.9%, respectively [1].

In endemic areas, patients with typhoid are considered as outpatients and treated with oral antibiotics, with hospital admission required only for complicated cases. Important concerns about typhoidal cases include relapse, which may complicate the illness; and fecal carriage, which can become chronic and may lead to continued transmission [2]. In late 1980s, 2–3 weeks of chloramphenicol was the treatment of choice for typhoid fever [3]. Subsequently, an increased number of strains with plasmid-mediated multidrug resistance (MDR) to chloramphenicol, ampicillin and cotrimoxazole are reported. Fluoroquinolone (ciprofloxacin and ofloxacin) has become the favored drug following the emergence of strains with MDR [4]. Although fluoroquinolones are superior to cephalosporins, the spread of strains with decreased susceptibility to ciprofloxacin has limited their effectiveness, particularly in Asia [5]. Extended spectrum cephalosporins (ceftriaxone and cefixime) and azithromycin are suitable alternatives for reduced fluoroquinolone susceptible *S.* Typhi [2,6]. The combinations of cephalosporin and azithromycin are quite frequently used to treat the patients who failed to respond promptly. The general rationale for their use is for broadening the spectrum of antimicrobial activity, to exploit the potential synergy between the drugs and reduce the probability of resistance development during course of treatment [7,8].

Although typhoid continues to be seen in large numbers, documented typhoid cases are reducing in recent years. The definitive diagnosis of typhoid fever requires a confirmed diagnosis based on the blood or bone marrow culture. However, blood culture has several limitations including amount of blood required (2–4 ml from toddlers and 10–15 ml from adolescents and adults) due to low levels of bacteremia and prior antibiotic use [9]. Furthermore, such laboratory facilities are limited or nonexistent in many Asian countries. An accurate and rapid diagnosis of typhoid fever improves the management of patients with appropriate antibiotic therapy. The sensitivity of blood culture is estimated to be between 40 and 60% [10]. Partly, this could be due to increased use of oral antibiotics prescribed in the communities without laboratory investigations.

This review summarizes the challenges in the laboratory diagnosis of typhoid fever and the current situation of MDR *S.* Typhi, and further explores the problem of antimicrobial resistance in these infections, management issues in clinical practice and combination treatment options for typhoidal *Salmonella*.

## Challenges in detection of typhoidal *Salmonella*

Although there is a decline in the incidence of *S.* Typhi, the true isolation of *S.* Typhi from blood cultures is still challenging [11]. Patients who are suspected of typhoid fever are given antimicrobials in the community and later referred to the tertiary care hospital for blood cultures. In India, ofloxacin-based combinations such as ofloxacin plus cefixime or ofloxacin plus azithromycin are most abundantly used and are available over the counter. According to the Center for Disease Dynamics, Economics & Policy, the consumption rate of cephalosporin has increased from 1887 to 7269 standard units/1000 people from 2000 to 2014, respectively. Similarly, macrolide usage has increased from 1166 to 1862 standard units/1000 people from 2000 to 2014, respectively. Meanwhile, fluoroquinolone usage ranged about 2608 to 2661 standard units/1000 population from 2000 to 2014, respectively [12]. This indicates the abundant usage of cephalosporins, fluoroquinolones and macrolides in the Indian setting. Owing to this, patients who have taken a cephalosporin-based combination (cefixime plus ofloxacin) are likely to exhibit a false-negative blood culture. It is, therefore, imperative to obtain a blood culture before initiating the antimicrobial therapy as the presence of antimicrobials could reduce the yield of *S.* Typhi in the blood cultures. To counteract this, commercial blood culture systems have incorporated synthetic resin molecules in the bottles to neutralize the antimicrobials present. Previous studies have demonstrated the neutralization of antimicrobials by the use of resin-based culture bottles [13–15]. However, certain antimicrobials have not been neutralized by the resins, hence this remains a limitation. This includes the fluoroquinolones and cephalosporins, which are the commonly used drugs in the community for suspected typhoid fever. Importantly, no published studies on the recovery of *S.* Typhi in blood cultures collected from patients with prior antibiotics were found.

Diagnosis of typhoid fever by conventional blood culture is challenging and time consuming as it takes about 24–48 h, after which the culture bottle flags positive. Although it is the gold standard method for detection of *S.* Typhi in the blood, turnaround time plays a significant role in the management. WIDAL slide agglutination test is the second most commonly prescribed test. Yet, poor sensitivity and specificity is a limitation [16]. Due to this, commercial rapid diagnostic tests (RDTs) are of great interest. However, owing to the poor sensitivity and specificity rates, definite detection is still limited. Typhidot-M, TUBEX-TM and Test-it are the three serological-based tests that have been evaluated. Performance of these tests have been shown to be poor and variable due to the high rates of disease burden in Asia, which is endemic for typhoid. In contrast, evaluation done in the Philippines has shown high sensitivity and specificity rates [17]. Among the three tests, TUBEX performance was found better with 78% of sensitivity and 87% specificity [18]. Due to these limitations, the WHO has issued no recommendations on the use of commercial RDTs [16]. Although RDT uses serum and urine samples, other than blood, isolation of *S.* Typhi from blood culture and bone marrow still remains the gold standard, as the susceptibility profile of the infected organisms cannot be inferred from serologic-based tests.

## MDR *Salmonella* Typhi

Approximately 65% MDR rates were seen during 1990–1992 in India, with 71% MDR rates in central India and 55% in southern parts of India [19]. Due to the high rates of MDR strains, the use of ampicillin, chloramphenicol and cotrimoxazole has declined, resulting in the increased use of fluoroquinolones (ofloxacin and ciprofloxacin) [20,21]. This has resulted in the phenomenon of decreased ciprofloxacin susceptibility (DCS) while cephalosporin resistance has begun to rise. Recently, MDR rates have drastically declined and the rates have fallen down from 26% in 2004 to 1% at present [20]. A similar picture is noted in Christian Medical College (CMC), Vellore, India, with less than 1% MDR rates (V. Balaji, Unpublished Data) and at New Delhi [10] and all over India. Furthermore, the Indian Network for Surveillance of Antimicrobial Resistance conducted between 2008 and 2010 has reported less than 5% of MDR S. Typhi across India [22]. In addition, the burden of antimicrobial resistance reported in the Indian studies are summarized in Table 1. The antimicrobial resistance mechanisms seen in S. Typhi and reported in the literature are mentioned in Table 2.

**Table 1.** **Burden of antimicrobial resistance rates reported in typhoidal *Salmonella* from Indian studies of past 15 years.**

**Antibiotic**	**Salmonella Typhi**	**Salmonella Paratyphi A**
Ampicillin^†^	5–72%	0–74%

Chloramphenicol^†^	3–27%	0–23%

Cotrimoxazole^†^	2.3–35%	0–36%

Nalidixic acid	78–100%	63–100%

Ciprofloxacin^‡^	0–97%	0–100%

Ceftriaxone	0–4%	0–6%

^†^MDR *S.* Typhi classified based in resistance to ampicillin, chloramphenicol and cotrimoxazole is coming down in the recent years.

^‡^High rates of ciprofloxacin resistance is due to the revised breakpoints in the CLSI guideline.

Refs: [23–29]

CLSI: Clinical and Laboratory Standards Institute; MDR: Multidrug-resistant.

**Table 2.** **Antimicrobial resistance mechanisms reported in typhoidal *Salmonella*.**

**Antibiotic class**	**Resistance mechanism**	**AMR genes responsible**	**Ref.**
ß-lactams (ampicillin)	Enzymatic hydrolysis	*bla*_TEM_, *bla*_CTX-M_, *bla*_SHV_	[30]

Chloramphenicol	Enzymatic hydrolysis	*cat*	[30]

Sulfonamides (trimethoprim/sulfamethoxazole)	Enzymatic hydrolysis	*dfr*A, *sul1* and *sul2*	[30]

Quinolones (nalidixic acid, ciprofloxacin, pefloxacin)	Drug target alterations (QRDR)	*gyr*A, *gyr*B, *par*C,*parE*	[31]

	Enzymatic hydrolysis (PMQR)	*qnr*A, *qnr*B, *qnr*S, *aac(6′)-Ib-cr, qepA*	[32]

Cephalosporins (ceftriaxone, cefixime)	AmpC ß-lactamases ESBLs	*bla*_CMY_, *bla*_FOX_ *bla*_CTX-M-15_	–

Macrolides (azithromycin)	Enzymatic hydrolysis overexpression of efflux pumps	*ereA, ereB, ermB, mefA, mphA, mphB* and *mphD*	[32–35]

ESBL: Extended spectrum beta-lactamase; PMQR: Plasmid-mediated quinolone resistance; QRDR: Quinolone-resistance determining region.

## Fluoroquinolones

Fluoroquinolones are a class of broad-spectrum antibiotic and are the direct inhibitors of bacterial DNA synthesis. Ciprofloxacin and ofloxacin are currently the drugs of choice for most cases of typhoid fever. These fluoroquinolones have become affordable for use in many resource-limited areas. Ofloxacin was an excellent choice of antibiotic used for treating typhoidal *Salmonella*, as it has excellent plasma and intracellular penetrations with more bactericidal activity [36]. However, the phenomenon of reduced susceptibility to fluoroquinolones has also been implicated in ofloxacin treatment. For the isolates with an MIC of ≥0.25 μg/ml, despite prolonged duration and increased dose of therapy, clinical failure has been seen [37]. This was observed with a randomized control trial (RCT) carried out between 1992 and 2001. The study provided evidence for the clinical failure cases of *S.* Typhi when the isolates ofloxacin MIC was ≥0.25 μg/ml. However, for isolates with an MIC of ≤0.125 μg/ml, 96% clinical success was observed [37]. Due to the inconsistencies in the cutoffs, the Clinical and Laboratory Standards Institute (CLSI, M100-S23) has removed the disc diffusion breakpoint criteria and recommended MIC testing for determining susceptibility, and revised the susceptible range interpretation from ≤2 μg/ml in 2012 to ≤0.12 μg/ml in 2013 for ofloxacin and levofloxacin as well [38].

For ciprofloxacin, the CLSI has revised the breakpoints for categorizing the clinical isolates ≤0.06 μg/ml as susceptible in 2012 [39]. Clinically, DCS is associated with clinical failure when ciprofloxacin MIC was ranging from 0.12 to 1 μg/ml, which is commonly seen in India [23,40]. MDR isolates with decreased ciprofloxacin susceptibility strains in India are on the increase, and this needs continuous monitoring [41]. Earlier, this was missed as the dependence was on nalidixic acid resistance (NAR) using disc diffusion testing. Moreover, monitoring NAR was not carried out with ciprofloxacin MIC.

However, in recent times, rising DCS with the sustained decrease in the MDR typhoidal *Salmonella* has been seen [20]. Similarly, Singhal *et al*. reported the declining MDR rates with the increased incidence of nalidixic acid-resistant, DCS isolates in Northern India [23]. There has been a reported decline in MDR with a parallel increase in DCS among *S.* Typhi. Ampicillin, chloramphenicol or cotrimoxazole are less likely preferred because of longer duration of therapy, threat of re-emergence of resistance, side effects and higher relapse rates.

Quinolone resistance is due to mutations in the quinolone-resistance determining regions of chromosomal genes such as *gyrA*, *gyrB*, *parC* and *parE* and plasmid-mediated *qnr, qepA and aacs(6′)-Ib-cr* genes [42]. Studies have reported that plasmid-mediated resistance showed reduced susceptibility to ciprofloxacin (MIC of 0.125–1.0 μg/ml), which could not be picked up by the nalidixic acid test [43]. Therefore, the CLSI and EUCAST recommend a new screening surrogate marker of pefloxacin (5 μg) disc diffusion for detecting both chromosomal- and plasmid-mediated resistance. This observation was evidenced by testing of pefloxacin, wherein 80% of the NAR and ciprofloxacin moderately susceptible isolates were resistant to pefloxacin [44,45]. Therefore, pefloxacin testing would ultimately help in the accurate identification of quinolone susceptibility for a better therapeutic success rate.

Gatifloxacin is an inexpensive fluroquinolone antibiotic with an excellent *in vitro* activity against *S.* Typhi. Gatifloxacin has the lowest MIC with the MIC_50_ of 0.19 μg/ml against *S.* Typhi, compared with ciprofloxacin and ofloxacin (MIC_50_, 0.75 μg/ml) [46]. Strikingly, gatifloxacin showed activity against isolates with DCS phenomenon. This is due to the ability of gatifloxacin to evade the point mutations in *gyrA* (Ser 83 Phe, Asp 87 Gly) contributing for DCS. For isolates included from India, the MIC range was observed to be 0.006–0.25 and 0.012–0.19 μg/ml for ciprofloxacin and gatifloxacin, respectively [47]. Furthermore, two trials have reported that good clinical response was noted with 7 days of therapy (10 mg/kg per day) and suggested gatifloxacin to be more effective than other agents. More importantly, fever clearance time was 92 h for gatifloxacin, while it was 138 h for patients who received cefixime, with 3.5 and 37.6% treatment failure in gatifloxacin and cefixime groups, respectively [46,48]. Dysglycemia is a major concern, for which sugar monitoring is essential [49]. Although gatifloxacin has an advantage over other quinolones, its use is banned in India.

## Cephalosporins

The cephalosporins are a class of ß-lactam antibiotics. Cephalosporins bind to the penicillin-binding proteins on bacteria and inhibit synthesis of the bacterial cell wall leading to cell lysis and death. With the rising DCS phenomenon, third-generation cephalosporins such as cefixime and ceftriaxone are preferred for the treatment of MDR and nalidixic acid-resistant isolates, to avoid clinical failures. As there is an increased use of cephalosporins, emergence of resistance is being observed [50–52]. Subsequently, 113 blood cultures confirmed that ceftriaxone-resistant *S.* Typhi cases have been reported from Karachi, Pakistan [53]. The mechanism of resistance is due to the production of ESBLs such as *bla*_SHV_ and *bla*_CTX-M-15_. Prospective clinical trials assessment in Asia showed that the resistance prevalence to ceftriaxone was 0%, with cure rate of 72–97% with 3–14 days of therapy. However, the relapse rate was about 0–17%, which occurred in patients treated for 7 days or less, while treatment for 8–14 days did not show any relapses [54]. Although the route of administration of ceftriaxone is parental, it requires hospitalization and increase in healthcare costs. Cefixime is the preferred choice in developing countries due to the availability of an oral form for uncomplicated typhoid fever.

## Azithromycin

Azithromycin, a member of the macrolide class of antibiotic, is an effective and convenient alternative for treating mild-to-moderate enteric fever. Clearly defined MIC breakpoints for azithromycin susceptibility have not been established, but data suggest that isolates with an MIC ≤16 μg/ml generally respond well to azithromycin and can be considered susceptible. The available clinical or pharmacokinetic/pharmacodynamic (PK/PD) data on azithromycin are limited. Notably, azithromycin nonsusceptibility rate was lower than the other agents. In Asia, analysis of 20 prospective clinical trials has proven that the relapse rate was 0% with azithromycin treatment, while the cure rate was about 81–100% with 5–7 days of therapy [55]. A study from New Delhi reported the MIC_90_ for *S.* Typhi and *S.* Paratyphi to be 24 μg/ml [40]. Following this, 34 and 38% resistance to azithromycin was reported in *S.* Typhi and *S*. Paratyphi A, respectively [55]. Recently, a case of azithromycin clinical and microbiological failure with the MIC of 4 μg/ml was reported from a patient with *S.* Typhi infection, from India [56]. Similarly, *S*. Paratyphi A with azithromycin clinical failure case with MIC of 12 μg/ml was reported in Australia [57]. Azithromycin is a valuable therapeutic alternative, due to higher intracellular concentrations (100-fold higher than in serum), early fever clearance rate (4–5 days), lower rates of relapse or reinfection and 5–7 days of therapy for complete cure [58]. However, azithromycin is not preferred as a monotherapy in severe typhoid, as it results in poor clinical response. Moreover, determination of MIC for azithromycin and monitoring clinical success is essential.

## Typhoid fever therapy: challenges in clinical practice

For the clinical management of typhoid fever, an early initiation of effective antimicrobial therapy shortens the duration of illness, and reduces the complications and mortality. The emergence and sustained circulation of reduced fluoroquinolone susceptible *S.* Typhi, the steady rise in nalidixic acid-resistant *S.* Typhi (NARST) and cases of azithromycin treatment failure are of concern [37,56]. Typically, in these cases, a slow resolution of fever and increased risk of clinical and microbiological failure were described [59–62]. Fluoroquinolones are commonly used for treating of MDR *S.* Typhi causing uncomplicated enteric fever. The widespread use of fluoroquinolones in primary healthcare settings contributes to emergence of strains with elevated MIC to ciprofloxacin and ofloxacin across Asia and parts of Africa [63,64]. The preferred therapy for management of typhoid fever is mentioned in Table 3.

**Table 3.** **Preferred therapy for management of typhoid fever.**

**Antimicrobial agents**	**Route of administration**	**Children**	**Adult**
Ceftriaxone	IM/IV	50 mg/kg per day IV; for 7–10 days	1–2 g per day IV; for 7–10 days

Ciprofloxacin, levofloxacin or other FQ^†^	Oral/IV	–	FQ given in full doses as recommended; for 7–10 days

Azithromycin	Oral	Used in complicated cases	500 mg twice a day for 5 days

Cefixime–ofloxacin	Oral	–	200–200 mg; for 7–14 days

^†^High-dose therapy is based on antimicrobial susceptibility profile of the infected typhoidal Salmonella strain, as majorities are nonsusceptible to quinolones. Least preferred as majority of the isolates show intermediate resistance to quinolones.

FQ: Fluoroquinolone; IM: Intramuscular; IV: Intravenous.

In addition to preventing weight-based dose titrations, most of these formulations also contain subtherapeutic doses of azithromycin. A review of 22 different Indian brands of fixed-dose combinations (FDCs) containing azithromycin with ofloxacin or cefixime revealed that while formulations contained an adequate fluoroquinolone or cephalosporin dose, the dose of azithromycin was 250 mg/tablet. This quantity is one fifth of the WHO recommended dose for a 60 kg individual (1200 mg; 10 mg/kg per day). Administering five tablets of the above FDC to a 60 kg individual would not only result in a high pill burden and poor adherence rates but also in toxic doses of the accompanying drug.

Despite Association of Physicians of India expert advisory panel for typhoid and WHO recommendations on weight-based antimicrobial dose titration, it is possible that typhoid patients continue to be prescribed antibiotics at standard doses. For azithromycin, while dose recommendations vary between 10 and 20 mg/kg per day for 5–7 days, specific weight-based dose and therapy-duration guidelines are yet to be determined. A randomized controlled trial by Parry *et al*. comparing ofloxacin (20 mg/kg body weight per day for 7 days), azithromycin (10 mg/kg per day for 7 days) and ofloxacin (15 mg/kg per day for 7 days) combined with azithromycin (10 mg/kg per day for the first 3 days) in MDR and NARST typhoid showed that defervescence time for azithromycin-treated patients was significantly shorter than that for patients treated with ofloxacin–azithromycin and ofloxacin alone. Table 4 summarizes the studies on monotherapy and combination therapy carried out for typhoid fever management.

**Table 4.** **Controlled and noncontrolled trials on monotherapy and combination therapy for typhoid fever.**

**Antibiotics**	**No. of patients (n)**	**Study type (duration of the study)/location**	**Dose/duration of therapy (days)**	**Fever defervescence (days)**	**Clinical/microbiological failure (%)**	**Relapse (%)**	**Fecal carriage (%)**	**Ref.**
Azithromycin vs ceftriaxone (IV; short course therapy)	108 (children)	Prospective study (2000)/Egypt	Azithromycin 10 mg/kg per day for 7 days 2.5 g per day for 7 days	Azithromycin – 4.1 Ceftriaxone – 3.9	*Clinical failure*: Azithromycin – 0 Ceftriaxone – 19 *Microbiological failure*: Azithromycin – 0 Ceftriaxone – 3	Azithromycin – 0 Ceftriaxone – 13	NA	[65]

Azithromycin vs ceftriaxone (IV; short course therapy)	149	Prospective study (2004)/Egypt	Azithromycin 20 mg/kg per day for 5 days 2.5 g per day for 5 days	Azithromycin – 4.5 Ceftriaxone – 3.6	*Clinical failure*: Azithromycin – 6 Ceftriaxone – 3 *Microbiological failure*: Azithromycin – 3 Ceftriaxone – 3	Azithromycin – 0 Ceftriaxone – 17	NA	[66]

Azithromycin	117 (children)	Open-labeled noncomparative study (2011)/India	Azithromycin – 20 mg/kg per day for 6 days	3.45	*Clinical failure*: Azithromycin – 6 *Microbiological failure*: Azithromycin – 15.5	NA	NA	[67]

Azithromycin vs ofloxacin	40 (adult)	Prospective (2012)/India	Ofloxacin – 200 mg orally twice daily for 7 days Azithromycin – 1 g on day 1 and then 500 mg daily from days 2–6	Ofloxain – 3.68 Azithromycin – 3.65	*Clinical failure*: Ofloxacin–10 Azithromycin – 0	No relapse	0	[68]

Ofloxacin vs cefixime	82 (children)	Randomized open trial (1995–1996)/Vietnam	Ofloxacin – 10 mg/kg per day for 5 days Cefixime – 20 mg/kg per day for 7 days	Ofloxacin – 4.4 Cefixime – 8.5	*Clinical failure*: Ofloxacin – 3 Cefixime – 25	Ofloxacin – 0 Cefixime – 3	Ofloxacin – 0 Cefixime – 3	[69]

Ofloxacin	235	Randomized open study/Vietnam	Ofloxacin (10 mg/kg per day) – 2 days Ofloxacin (10 mg/kg per day) – 3 days	2-day group: 3.8 3-day group: 4.2	*Clinical failure:* 2-day group: 13.5 3-day group: 7.5 *Microbiological failure:* 2-day group: 4 3-day group: 1	2-day group: 2 3-day group: 2	NA	[70]

Chloramphenicol vs ofloxacin	50	Randomized open study/Laos	Chloramphenicol (50 mg/kg per day) for 14 days Ofloxacin (15 mg/kg per day) – 3 days	Chloramphenicol – 3.7 Ofloxacin – 2.2	*Clinical failure:* Chloramphenicol – 1%	NA	NA	[71]

Ofloxacin vs azithromycin	88	Randomized control study/Nepal	Azithromycin (20 mg/kg per day) for 5 days Ofloxacin – (8 mg/kg per day) for 5 days	Ofloxacin – 7 Azithromycin – 5	*Clinical failure:* Ofloxacin – 13.6 Azithromycin – 4.5 *Microbiological failure:* Ofloxacin – 4.5 Azithromycin – 0	Ofloxacin – 4.5 Azithromycin – 0	Ofloxacin – 41 Azithromycin – 0	[72]

Ofloxacin, azithromycin, ofloxacin/azithromycin	187	Randomized control study (1998–2002)/Vietnam	Ofloxacin – 20 mg/kg per day for 7 days Azithromycin – 10 mg/kg per day for 7 days Ofloxacin (15 mg/kg per day)/azithromycin (10 mg/kg per day)	Ofloxacin – 8.2 Azithromycin – 5.8 Ofloxacin/azithromycin – 7.1	*Clinical failure:* Ofloxacin – 12.3 Azithromycin – 18.8 Ofloxacin/azithromycin – 6.4 *Microbiological failure:* Ofloxacin – 1.6 Azithromycin – 0 Ofloxacin/azithromycin - 1.6 (patient experienced microbiologcal failure)	Relapse was not seen in patients treated with ofloxacin or azithromycin or ofloxacin/azithromycin	Ofloxacin – 19.4 Azithromycin – 1.6 Ofloxacin/azithromycin – 6.5	[73]

Gatifloxacin vs azithromycin	287	RCT/Vietnam	Gatifloxacin (10 mg/kg per day) for 7 days Azithromycin (20 mg/kg per day) for 7 days	Gatifloxacin – 4.4 Azithromycin – 4.4	*Clinical failure:* Gatifloxacin – 4.3 Azithromycin – 4.2 *Microbiological failure:* Gatifloxacin – 1.4 Azithromycin – 2.2	Gatifloxacin – 2.9 Azithromycin – 0	Gatifloxacin – 0.7 Azithromycin – 0	[46]

Gatifloxacin vs cefixime	390	RCT/Nepal	Gatifloxacin (10 mg/kg per day) for 7 days Cefixime (20 mg/kg per day) for 7 days	Gatifloxacin – 3.8 Cefixime – 5.7	*Clinical failure:* Gatifloxacin – 1 Cefixime – 27	Gatifloxacin – 0.9 Cefixime – 3	NA	[48]

Gatifloxacin vs chloramphenicol	844	RCT/Nepal	Gatifloxacin (10 mg/kg per day) for 7 days Chloramphenicol (75 mg/kg per day) for 14 days		*Clinical failure:* Gatifloxacin – 7 Chloramphenicol – 8 *Microbiological failure:* Gatifloxacin – 1 Chloramphenicol – 0	Gatifloxacin – 2 Chloramphenicol – 4	NA	[74]

Gatifloxacin vs ofloxacin	627	RCT/Nepal	Gatifloxacin (10 mg/kg per day) for 7 days Ofloxacin (10 mg/kg per day) for 7 days	Gatifloxacin – 3.3 Ofloxacin – 4.7	C*linical failure*: Gatifloxacin – 5 Ofloxacin – 7 *Microbiological failure:* None	Gatifloxacin – 5 Ofloxacin – 6	NA	[75]

Gatifloxacin vs ceftriaxone	239	RCT/Nepal	Gatifloxacin (10 mg/kg per day) for 7 days Ceftriaxone (2 g per day) for 7 days	Gatifloxacin – 2.43 Ceftriaxone – 2.93	Gatifloxacin – 15 Ceftriaxone – 16	NA	NA	[76]

Azithromycin–ceftriaxone combination	25	Prospective study (2014–2015)/India	Ceftriaxone (IV) – 2 g daily for 14 days Azithromycin – 500 mg daily for the first 7 days	4.8	Clinical and microbiological failure: 4	No relapse	NA	[77]

IV: Intravenous; NA: Not available; RCT: Randomized clinical trial.

Fluoroquinolones and azithromycin attain better tissue penetration and kill bacteria within monocytes/macrophages more efficiently compared with third-generation cephalosporins, which primarily achieve blood stream bacterial clearance. This fact has resulted in the exploration of the possibility of combination regimens in typhoid treatment. Studies demonstrating combination therapy superiority compared with monotherapy are, however, limited. A study of 37 individuals with NARST *S. paratyphi* A bacteremia demonstrated that the time to fever defervescence was shorter in patients treated with ceftriaxone–azithromycin combination therapy compared with those treated with ceftriaxone monotherapy [78]. However, the significance of this finding should be confirmed with large clinical trials for its safety and efficacy. In contrast, a comparative Indian study of 62 typhoid fever patients failed to reveal a significant difference in time to defervescence between the single drug (fluoroquinolone or third-generation cephalosporin) and combination drug group (fluoroquinolone with third-generation cephalosporin) [79].

## Concern in management in developing countries

In South East Asia (SEA) and Africa, a rise in the MIC of fluoroquinolones against *S.* Typhi has shown a significant increase and is associated with longer duration of fever defervescence [37,64,80]. This reiterates that fluoroquinolone should not be recommended for empirical treatment of typhoid fever. Azithromycin treatment failure and emergence of cephalosporin resistance are of concern [52,56,81]. However, antimicrobial resistance to ceftriaxone/cefixime and azithromycin remains rare. In the areas where fluoroquinolone resistance is uncommon, the quinolones are the treatment of choice for all the age groups. Treatment at the maximal recommended doses (20 mg/kg per day) for 7–10 days has been successful in 90–95% of patients [82]. Remarkably, quinolone-resistant *S.* Typhi strains are on rise and therefore the drug of choice is limited to azithromycin or cephalosporins, which are expensive.

Randomized control trials (RCTs) of third-generation cephalosporins, primarily ceftriaxone/cefixime, in treating typhoid fever have reported an average fever clearance of 7 days, with a treatment failure rate between 5 and 10%, relapse rates of 3–6% and fecal carriage of less than 3% [48,65,69,76]. Similarly, RCTs on treating uncomplicated with azithromycin for 5–7 days resulted in 95% cure rate and fever defervescence of 4–6 days [46,66–68,72]. Notably, relapse rate and fecal carriage are less than 3%. However, treatment with cephalosporin exhibits a slow response with a mean time of 5–7 days or an even longer duration of fever defervescence [83]. This could be attributed to poor penetration capability of the drug into cells, and thus difficulty to eradicate the bacteria from the intracellular niche. An approach of cephalosporin–azithromycin combination therapy has been suggested based on the individual pharmacokinetics [77]. The complimentary action of cephalosporins on the extracellular compartment and azithromycin on the intracellular compartment could be beneficial in treating *S.* Typhi infection.

Fixed dose combinations (FDCs) of cefixime–ofloxacin (200–200 mg) are widely used across India. With the biggest market sale of approximately $46 million, they are increasingly available as over-the-counter prescription. An *in vitro* study has reported that none of the tested *S.* Typhi isolates demonstrated antagonism against this combination [84]. Some FDCs are banned for clinical use in India; these include cefixime–azithromycin, cefpodoxime–azithromycin and ofloxacin–azithromycin. Azithromycin is known to reduce cefpodoxime efficacy by PD antagonism [85]. The expert committee of the Chief Drug Advisory body of India has deemed that these FDCs are irrational. Currently, two clinical trials (clinicaltrial.gov: NCT02224040, NCT02708992) using cefixime–azithromycin and ceftriaxone–azithromycin for various indication are in progress. In the differential diagnosis of febrile illness, doxycycline plus cefixime–azithromycin is prescribed as empiric therapy, especially in SEA where rickettsial infection is common. However, drug–drug interactions showed that doxycycline decreases effects of ceftriaxone by PD antagonism [85]. There is a huge market available for these combinations in SEA and they are frequently prescribed for pelvic inflammatory infection. Greater misuse is expected with availability of this cephalosporin–azithromycin combination, which is worrisome.

Collectively, this reiterates that in the context of reduced fluoroquinolone susceptibility, third-generation cephalosporins, such as ceftriaxone/cefixime or azithromycin, are the treatment of choice for treating *S.* Typhi in developing countries. In addition, evidence from the progressing clinical trials (clinicaltrial.gov: NCT02224040, NCT02708992), on cefixime–azithromycin and ceftriaxone–azithromycin FDCs could prove them to be be an effective alternative therapy for the management of quinolone-resistant *S.* Typhi.

## Natural products in treating typhoid fever

The use of medicinal plants to treat disease is almost universal and is more affordable than purchasing expensive conventional drugs. Natural plants contain phytoconstituents containing chemical properties similar to synthetic antibiotics. Since the issues with antibiotic efficacy of monotherapy have been reported, it has become essential to evaluate biological properties of plants. Tulsi (*Ocimum sanctum*) has potent antibacterial activity against *S.* Typhi [FL1] [86]. An *in vitro* study has demonstrated significant synergy of chloramphenicol and trimethoprim with *O. sanctum* leaf extract against *S.* Typhi [86]. Similarly, acetone mango leaf extract was demonstrated with the inhibitory effect at the concentration of 10–50 μg/ml against MDR *S.* Typhi [87]. Nkuo-Akenji *et al*. have reported the lowest MIC of 0.02–0.06 μg/ml against *S.* Typhi with the formulation of the methanol leaf extract of *Cymbogogon citratus*, *Carica papaya* and *Zea mays* silk [88,89]. Overall, studies have reported potent *in vitro* activity of various leaf extracts against *S.* Typhi. However, *in vivo* studies on the bioavailability, PK/PD, antibacterial interaction or safety profile of these natural products are limited [90]. Consequentially, defined dosage and its significant activity in treating complicated typhoid fever remain uncertain.

## Conclusion

Given the emergence of fluoroquinolone and azithromycin nonsusceptible *S.* Typhi strains, it is interesting to note that there has been a recent sustained decrease in MDR typhoidal *Salmonella* isolates in India. This observation suggests the possible reintroduction of ampicillin, chloramphenicol and cotrimoxazole as first-line options. While these agents are inexpensive and easily available, disadvantages associated with their use include a longer duration of therapy to prevent relapses, higher relapse rates and serious adverse effects. The rate and likelihood of resistance to an antimicrobial is a function of the frequency of its use, due to the excessive selective pressure generated. It, therefore, appears likely that the decline in MDR rates would not be sustained if the abovementioned traditional first-line drugs were to be recruited into routine first-line use.

In severe cases of typhoid fever, ceftriaxone IV (2 g per day) for 10–14 days is given followed by azithromycin (20 mg/kg per day) for 7 days. For DCS *S.* Typhi causing uncomplicated typhoid fever, fluoroquinolone therapy is to be avoided. If necessary, higher dose of ciprofloxacin (20 mg/kg per day) may be more effective in treating typical cases of mild typhoid fever. However, with fluoroquinolone therapy, delay in fever defervescence, relapse and faecal carriage can be expected. Therefore, treatment with azithromycin (20 mg/kg per day) for 7 days is recommended for treating uncomplicated cases.

## Future perspective

Increased use of cephalosporin or azithromycin in treating fluoroquinolone nonsusceptible *S.* Typhi may lead to a rise in cephalosporin resistance or azithromycin treatment failure. In the era of MDR, combination therapy could be an alternative for treating *S.* Typhi infection. The goal of this article is to inspire more research into the FDCs available for treatment of *S.* Typhi. *In vitro* combination testing of cefixime–azithromycin and ceftriaxone–azithromycin will provide detailed insights into the antimicrobial effect and drug–drug interaction of these combinations. The result of on-going clinical trials is welcomed and this could be an added value for research output. Furthermore, the extensive studies on the bioavailability, dosing, antibacterial effect, PK/PD and safety profile of natural products could reveal an alternative for antibiotics in treating *S.* Typhi infection.

**Summary points**

- Reduced fluoroquinolones susceptibility of *Salmonella enterica* subsp. *enterica* serovar Typhi (*S.* Typhi) is on the rise and of significant concern in developing countries.
- Third-generation cephalosporins (cefixime, ceftriaxone) or azithromycin are the alternative for treating *S.* Typhi infection with decreased susceptibility to ciprofloxacin.
- An approach of cephalosporin plus azithromycin can address the shortcomings of cephalosporins including poor response and longer duration for fever defervescence.
- Cefixime–ofloxacin combination is the hugely marketed fixed-dose combination in India, for the treating *S.* Typhi infection.
- Evidence from the on-going clinical trials (clinicaltrial.gov: NCT02224040, NCT02708992) could be value-added information for using fixed-dose combinations, as a standard practice in treating *S.* Typhi, especially in Asia and parts of Africa.

## References

[1] John J, Van Aart CJC, Grassly NC (2016). The burden of typhoid and paratyphoid in India: systematic review and meta-analysis. *PLoS Negl. Trop. Dis.*.

[2] Parry CM, Beeching NJ (2009). Treatment of enteric fever. *BMJ*.

[3] Kalra SP, Naithani N, Mehta SR (2003). current trends in the management of typhoid fever. *Med. J. ArMed. Forces India.*.

[4] Divyashree S, Nabarro LE, Veeraraghavan B (2016). Enteric fever in India: current scenario and future directions. *Trop. Med. Int. Health.*.

[5] Rahman BA, Wasfy MO, Maksoud MA (2014). Multi-drug resistance and reduced susceptibility to ciprofloxacin among *Salmonella enterica* serovar Typhi isolates from the Middle East and Central Asia. *New Microbes New Infect.*.

[6] Bhutta ZA (2006). Current concepts in the diagnosis and treatment of typhoid fever. *BMJ*.

[7] Beeching NJ, Parry CM (2011). Outpatient treatment of patients with enteric fever. *Lancet Infect. Dis.*.

[8] Parry CM, Basnyat B, Crump JA (2013). The management of antimicrobial-resistant enteric fever. *Expert Rev. Anti. Infect. Ther.*.

[9] World Health Organization, Department of Vaccines and Biologicals (2003). Background document: the diagnosis, treatment and prevention of typhoid fever. http://www.who.int/rpc/TFGuideWHO.pdf.

[10] Wain J, Hosoglu S (2008). The laboratory diagnosis of enteric fever. *J. Infect. Dev. Ctries*.

[11] Mogasale V, Ramani E, Mogasale VV (2016). What proportion of Salmonella Typhi cases are detected by blood culture? A systematic literature review. *Ann. Clin. Microbiol. Antimicrob.*.

[12] (2015). Center for Disease Dynamics, Economics & Policy, State of the World's Antibiotics, 2015. https://resistancemap.cddep.org/AntibioticUse.php.

[13] Flayhart D, Borek AP, Wakefield T (2007). Comparison of BACTEC PLUS blood culture media to BacT/Alert FA blood culture media for detection of bacterial pathogens in samples containing therapeutic levels of antibiotics. *J. Clin. Microbiol.*.

[14] Miller NS, Rogan D, Orr BL (2011). Comparison of BD Bactec Plus blood culture media to VersaTREK Redox blood culture media for detection of bacterial pathogens in simulated adult blood cultures containing therapeutic concentrations of antibiotics. *J. Clin. Microbiol.*.

[15] Mitteregger D, Barousch W, Nehr M (2013). Neutralization of antimicrobial substances in new BacT/Alert FA and FN Plus blood culture bottles. *J. Clin. Microbiol.*.

[16] Keddy KH, Sooka A, Letsoalo ME (2011). Sensitivity and specificity of typhoid fever rapid antibody tests for laboratory diagnosis at two sub-Saharan African sites. *Bull. World Health Organ.*.

[17] Kawano RL, Leano SA, Agdamag DM (2007). Comparison of serological test kits for diagnosis of typhoid fever in the Philippines. *J. Clin. Microbiol.*.

[18] Wijedoru L, Mallett S, Parry CM (2017). Rapid diagnostic tests for typhoid and paratyphoid (enteric) fever. *Cochrane Database Syst.*.

[19] Harish BN, Menezes GA (2011). Antimicrobial resistance in typhoidal salmonellae. *Indian J. Med. Microbiol.*.

[20] Balaji V, Sharma A, Ranjan P (2014). Revised ciprofloxacin breakpoints for *Salmonella Typhi*: its implications in India. *Indian J. Med. Microbiol.*.

[21] Raveendran R, Wattal C, Sharma A (2008). High level ciprofloxacin resistance in Salmonella enterica isolated from blood. *Indian J. Med. Microbiol.*.

[22] Joshi S (2012). Antibiogram of *S. enterica* serovar Typhi and *S. enterica* serovar Paratyphi A: a multi-centre study from India. *WHO South East Asia J. Public Health.*.

[23] Singhal L, Gupta PK, Kale P (2014). Trends in antimicrobial susceptibility of *Salmonella Typhi* from North India (2001–2012). *Indian J. Med. Microbiol.*.

[24] Dutta S, Das S, Mitra U (2014). Antimicrobial resistance, virulence profiles and molecular subtypes of Salmonella enterica serovars Typhi and Paratyphi A blood isolates from Kolkata, India during 2009–2013. *PLoS ONE*.

[25] Jain S, Chugh TD (2013). Antimicrobial resistance among blood culture isolates of *Salmonella enterica* in New Delhi. *J. Infect. Dev. Ctries.*.

[26] Shetty AK, Shetty IN, Furtado ZV (2012). Antibiogram of Salmonella isolates from blood with an emphasis on nalidixic acid and chloramphenicol susceptibility in a tertiary care hospital in coastal Karnataka: a prospective study. *J. Lab. Physicians*.

[27] Menezes GA, Harish BN, Khan MA (2012). Antimicrobial resistance trends in blood culture positive *Salmonella Typhi* isolates from Pondicherry, India, 2005–2009. *Clin. Microbiol. Infect.*.

[28] Muthu G, Suresh A, Sumathy G (2011). Studies on antimicrobial susceptibility pattern of Salmonella isolates from Chennai, India. *Int. J. Pharma. Bio. Sci.*.

[29] Bhattacharya SS, Das U, Choudhury BK (2011). Occurrence & antibiogram of Salmonella typhi & S. paratyphi A isolated from Rourkela, Orissa. *Indian J. Med. Res.*.

[30] Olarte J, Galindo E (1973). Salmonella typhi resistant to chloramphenicol, ampicillin, and other antimicrobial agents: strains isolated during an extensive typhoid fever epidemic in Mexico. *Antimicrob. Agents Chemother.*.

[31] Wong VK, Baker S, Pickard DJ (2015). Phylogeographical analysis of the dominant multidrug-resistant H58 clade of *Salmonella Typhi* identifies inter- and intracontinental transmission events. *Nat. Genet.*.

[32] Rodrigues C, Kapil A, Sharma A (2017). Whole-genome shotgun sequencing of cephalosporin-resistant *Salmonella enterica* Serovar Typhi. *Genome Announc.*.

[33] Akinyemi KO, Iwalokun BA, Oyefolu AO (2017). Occurrence of extended-spectrum and AmpC β-lactamases in multiple drug resistant Salmonella isolates from clinical samples in Lagos, Nigeria. *Infect. Drug Resist.*.

[34] Phoba MF, Barbé B, Lunguya O (2017). *Salmonella enterica* serovar Typhi producing Ctx-m-15 extended spectrum β-lactamase in the Democratic Republic of the Congo. *Clin. Infect. Dis.*.

[35] Sjölund-Karlsson M, Joyce K, Blickenstaff K (2011). Antimicrobial susceptibility to azithromycin among *Salmonella enterica* isolates from the United States. *Antimicrob. Agents Chemother.*.

[36] Bethell DB, Day NPJ, Nguyen MD (1996). Pharmacokinetics of oral and intravenous ofloxacin in multidrug-resistant typhoid fever. *Antimicrob. Agents Chemother.*.

[37] Parry CM, Vinh H, Chinh NT (2011). The influence of reduced susceptibility to fluoroquinolones in *Salmonella enterica* serovar Typhi on the clinical response to ofloxacin therapy. *PLoS Negl. Trop. Dis.*.

[38] Clinical and Laboratory Standards Institute (2013). Performance standards for antimicrobial susceptibility testing; twenty-third informational supplement. *Clinical and Laboratory Standards Institute*.

[39] Clinical and Laboratory Standards Institute (2012). Performance standards for antimicrobial susceptibility testing; twenty-second informational supplement. *Clinical and Laboratory Standards Institute*.

[40] Capoor MR, Nair D, Aggarwal P (2007). *Salmonella enterica* serovar typhi: molecular analysis of strains with decreased susceptibility and resistant to ciprofloxacin in India from 2001–2003. *Braz. J. Infect. Dis.*.

[41] Dahiya S, Sharma P, Kumari B (2017). A Characterisation of antimicrobial resistance in Salmonellae during 2014–2015 from four centres across India: an ICMR antimicrobial resistance surveillance network report. *Indian J. Med. Microbiol.*.

[42] Hopkins KL, Day M, Threlfall EJ (2008). Plasmid-mediated quinolone resistance in *Salmonella enterica*, United Kingdom. *Emerg. Infect. Dis.*.

[43] Balaji V, Shalini A, Dhiviya Prabaa MS (2016). Molecular characterization of intermediate susceptible Typhoidal *Salmonella* to ciprofloxacin and its impact. *Mol. Diagn. Ther.*.

[44] Balaji V, Shalini A, Dhiviya Prabaa MS (2016). Pefloxacin as a surrogate marker for fluoroquinolone susceptibility for *Salmonella* Typhi: problems and prospects. *J. Clin. Diagn. Res.*.

[45] Sharma P, Dahiya S, Kumari B (2017). Pefloxacin as a surrogate marker for quinolone susceptibility in *Salmonella enterica* serovars Typhi & Paratyphi A in India. *Indian J. Med. Res.*.

[46] Dolecek C, Tran TPL, Nguyen NR (2008). A multi-center randomised controlled trial of gatifloxacin versus azithromycin for the treatment of uncomplicated typhoid fever in children and adults in Vietnam. *PLoS ONE*.

[47] Chau TT, Campbell JI, Galindo CM (2007). Antimicrobial drug resistance of *Salmonella enterica* serovar typhi in asia and molecular mechanism of reduced susceptibility to the fluoroquinolones. *Antimicrob. Agents Chemother.*.

[48] Pandit A, Arjyal A, Day JN (2007). An open randomized comparison of gatifloxacin versus cefixime for the treatment of uncomplicated enteric fever. *PLoS ONE*.

[49] Zvonar R (2006). Gatifloxacin-induced dysglycemia. *Am. J. Health Syst. Pharm.*.

[50] Naveen Kumar DR, Dhiviya Prabaa MS, Baby Abirami S (2016). Draft genome sequence of blaTEM-1 mediated cephalosporin resistant *Salmonella* Typhi from blood stream infection. *J. Glob. Antimicrob. Resist.*.

[51] Rodrigues Camilla, Kapil Arti, Sharma Anita (2017). Whole-genome shotgun sequencing of cephalosporin resistant *Salmonella enterica* Serovar Typhi. *Genome Announc.*.

[52] Klemm EJ, Shakoor S, Page AJ (2018). Emergence of an extensively drug-resistant *Salmonella enterica* Serovar Typhi clone harboring a promiscuous plasmid encoding resistance to fluoroquinolones and third-generation cephalosporins. *MBio*.

[53] Yousafzai MT (4–6 April 2017). Outbreak investigation of cefriaxone resistant *S. Typhi* in Hyderabad, Pakistan. *10th International Conference on Typhoid and Other Invasive Salmonellosis*.

[54] Butler T (2011). Treatment of typhoid fever in the 21st century: promises and shortcomings. *Clin. Microbiol. Infect. Dis.*.

[55] Rai S, Jain S, Prasad KN (2012). Rationale of azithromycin prescribing practices for enteric fever in India. *Indian J. Med. Microbiol.*.

[56] Manesh A, Balaji V, Kumar DRN (2017). A case of clinical and microbiological failure of azithromycin therapy in *Salmonella enterica* serotype Typhi despite low azithromycin MIC. *Int. J. Infect. Dis.*.

[57] Fernando S, Molland JG, Gottlieb T (2012). Failure of oral antibiotic therapy, including azithromycin, in the treatment of a recurrent breast abscess caused by *Salmonella enterica* serotype Paratyphi A. *Pathog. Glob. Health.*.

[58] Parry CM, Thieu NT, Dolecek C (2015). Clinically and microbiologically derived azithromycin susceptibility breakpoints for *Salmonella enterica* serovars Typhi and Paratyphi A. *Antimicrob. Agents Chemother.*.

[59] Chiou CS, Alam M, Kuo JC (2015). Chromosome-mediated multidrug resistance in *Salmonella enterica* serovar Typhi. *Antimicrob. Agents Chemother.*.

[60] Hassing RJ, Goessens WH, Mevius DJ (2013). Decreased ciprofloxacin susceptibility in Salmonella Typhi and Paratyphi infections in ill-returned travellers: the impact on clinical outcome and future treatment options. *Eur. J. Clin. Microbiol. Infect. Dis.*.

[61] Koirala S, Basnyat B, Arjyal A (2013). Gatifloxacin versus ofloxacin for the treatment of uncomplicated enteric fever in Nepal: an open-label, randomized, controlled trial. *PLoS Negl. Trop. Dis.*.

[62] Parry CM, Thompson C, Vinh H (2014). Risk factors for the development of severe typhoid fever in Vietnam. *BMC Infect. Dis.*.

[63] Smith AM, Govender N, Keddy KH (2010). Group for Enteric, Respiratory and Meningeal Disease Surveillance in South Africa (GERMS-SA). Quinolone-resistant *Salmonella typhi* in South Africa, 2003–2007. *Epidemiol. Infect.*.

[64] Kariuki S, Revathi G, Kiiru J (2010). Typhoid in Kenya is associated with a dominant multidrug-resistant *Salmonella enterica* serovar Typhi haplotype that is also widespread in Southeast Asia. *J. Clin. Microbiol.*.

[65] Frenck RW, Nakhla I, Sultan Y (2000). Azithromycin versus ceftriaxone for the treatment of uncomplicated typhoid fever in children. *Clin. Infect. Dis.*.

[66] Frenck RW, Mansour A, Nakhla I (2004). Short-course azithromycin for the treatment of uncomplicated typhoid fever in children and adolescents. *Clin. Infect. Dis.*.

[67] Aggarwal A, Ghosh A, Gomber S (2011). Efficacy and safety of azithromycin for uncomplicated typhoid fever: an open label non-comparative study. *Indian Pediatr.*.

[68] Chandey M, Multani AS (2012). A comparative study of efficacy and safety of azithromycin and ofloxacin in uncomplicated typhoid fever: a randomised, open labelled study. *J. Clin. Diagn. Res.*.

[69] Cao XT, Kneen R, Nguyen TA (1999). A comparative study of ofloxacin and cefixime for treatment of typhoid fever in children. The Dong Nai Pediatric Center Typhoid Study Group. *Pediatr. Infect. Dis J.*.

[70] Vinh H, Duong NM, Phuong LT (2005). Comparative trial of short-course ofloxacin for uncomplicated typhoid fever in Vietnamese children. *Ann. Trop. Paediatr.*.

[71] Phongmany S, Phetsouvanh R, Sisouphone S (2005). A randomized comparison of oral chloramphenicol versus ofloxacin in the treatment of uncomplicated typhoid fever in Laos. *Trans. R. Soc. Trop. Med. Hyg.*.

[72] Chinh NT, Parry CM, Ly NT (2000). A randomized controlled comparison of azithromycin and ofloxacin for treatment of multidrug-resistant or nalidixic acid-resistant enteric fever. *Antimicrob. Agents Chemother.*.

[73] Parry CM, Ho VA, Phuong LT (2007). Randomized controlled comparison of ofloxacin, azithromycin, and an ofloxacin-azithromycin combination for treatment of multidrug-resistant and nalidixic acid-resistant typhoid fever. *Antimicrob. Agents Chemother.*.

[74] Arjyal A, Basnyat B, Koirala S (2011). Gatifloxacin versus chloramphenicol for uncomplicated enteric fever: an open-label, randomised, controlled trial. *Lancet Infect. Dis.*.

[75] Koirala S, Basnyat B, Arjyal A (2013). Gatifloxacin versus ofloxacin for the treatment of uncomplicated enteric fever in Nepal: an open-label, randomized, controlled trial. *PLoS Negl. Trop. Dis.*.

[76] Arjyal A, Basnyat B, Nhan HT (2016). Gatifloxacin versus ceftriaxone for uncomplicated enteric fever in Nepal: an open-label, two-centre, randomised controlled trial. *Lancet Infect. Dis.*.

[77] Giri VP, Giri OP, Srivastava A (2015). A clinical trial of treatment of uncomplicated typhoid fever: efficacy of ceftriaxone-azithromycin combination. *Int. J. Basic Clin. Pharmacol.*.

[78] Meltzer E, Stienlauf S, Leshem E (2014). A large outbreak of Salmonella Paratyphi. A infection among Israeli travelers to Nepal. *Clin. Infect. Dis.*.

[79] Balasubramanian S, Rajeswari, Sailakshmi, Shivbalan S (2006). Single vs multidrugtherapy in enteric fever. *Indian J. Pediatr.*.

[80] Thompson CN, Karkey A, Dongol S (2017). Treatment response in enteric fever in an era of increasing antimicrobial resistance: an individual patient data analysis of 2092 participants enrolled into 4 randomized, controlled trials in Nepal. *Clin. Infect. Dis.*.

[81] Munir T, Lodhi M, Ansari JK (2016). Extended spectrum beta lactamase producing cephalosporin resistant *Salmonella Typhi*, reported from Rawalpindi, Pakistan. *J. Pak. Med. Assoc.*.

[82] Parry CM, Hien TT, Dougan G (2002). Typhoid fever. *N. Engl. J. Med.*.

[83] Capoor MR, Nair D (2010). Quinolone and cephalosporin resistance in enteric fever. *J. Glob. Infect. Dis.*.

[84] Bakthavatchalam YD, Kumar DT, Tayubi IA (2017). *In vitro* efficacy and in silico analysis of cefixime-ofloxacin combination for *Salmonella Typhi* from bloodstream infection. *J. Appl. Microbiol.*.

[85] Preston LC (2015). Stockley's drug interactions. http://www.medicines-complete.com.

[86] Mandal S, Mandal MD, Pal NK (2012). Enhancing chloramphenicol and trimethoprim *in vitro* activity by Ocimum sanctum Linn. (Lamiaceae) leaf extract against *Salmonella enterica* serovar Typhi. *Asian Pac. J. Trop. Med.*.

[87] Hannan A, Asghar S, Naeem T (2013). Antibacterial effect of mango (Mangifera indica Linn.) leaf extract against antibiotic sensitive and multi-drug resistant *Salmonella* Typhi. *Pak. J. Pharm. Sci.*.

[88] Nkuo-Akenji T, Ndip R, McThomas A (2001). Anti-*Salmonella* activity of medicinal plants from Cameroon. *Cent. Afr. J. Med.*.

[89] Efunwole O, Adetuberu I, Oladipupo A (2018). Antibacterial effect of Carica papaya against *Salmonella* Typhi, causative agent of Typhoid fever. *IOSR-JESTFT*.

[90] Chattopadhyay D, Ojha D, Mukherjee H (2018). Validation of a traditional preparation against multi-drug resistant *Salmonella* Typhi and its protective efficacy in *S. Typhimurium* infected mice. *BioMed. Pharmacother.*.

